# Earthworm Powder Mitigates Soybean Meal-Induced Growth Inhibition in Rice Field Eel (*Monopterus albus*) by Regulating Appetite and Improving Intestinal Health

**DOI:** 10.3390/biology15060456

**Published:** 2026-03-11

**Authors:** Kaiwen Hou, Hui Wang, Lin Zhang, Xiaohong Wang, Hao Zhang, Fangling Wang, Qiaonan Deng, Xiangxiang Yang, Junzhi Zhang, Yi Hu

**Affiliations:** 1 College of Chemistry and Chemical Engineering, Central South University of Forestry & Technology, Changsha 410004, China; 2 Hunan International Joint Laboratory of Woody Biomass Conversion, Central South University of Forestry and Technology, Changsha 410004, China; 3 College of Materials and Energy, Central South University of Forestry and Technology, Changsha 410004, China; 4 Fisheries College, Hunan Agricultural University, Changsha 410128, Chinazhjun123@hunau.edu.cn (J.Z.)

**Keywords:** *Monopterus albus*, earthworm powder, soybean meal replacement, appetite regulation, gut microbiota

## Abstract

The substitution of fish meal with soybean meal (SBM) in aquafeeds aligns with sustainable development but often leads to depressed feed intake and growth in fish. This study aimed to investigate the mitigating effect of earthworm powder (EP) on these negative impacts in rice field eels (*Monopterus albus*), focusing on appetite regulation, intestinal health, and gut microbiota. Three isonitrogenous (~41% crude protein) and isolipidic (~6.4% crude lipid) diets (control [CON], high-SBM [SBM], and SBM + 2.5% EP [EP]) were tested in a 56-day trial. Juveniles (initial weight 18.00 ± 0.01 g) were stocked at 40 fish per net (0.5 m × 0.5 m× 0.5 m) and fed to visual satiety once daily. The results indicated that EP improved growth performance through a dual mechanism. Firstly, it was associated with significantly increased feed intake, correlated with the upregulated expression of orexigenic genes (*agrp*, *npy*) in the brain, and associated with reduced levels of anorexigenic hormones (Cholecystokinin, Leptin). Secondly, it correlated with enhanced intestinal health, evidenced by improved morphology (villus height, goblet cells), improved digestive enzyme activity, enhanced antioxidant capacity (increased Catalase and Superoxide Dismutase activities), repaired intestinal barrier function (upregulated *zo-1*, *cla-12*), and alleviated intestinal inflammation (downregulated *tnf-α*, *il-1β*). Furthermore, EP supplementation was associated with a shift in gut microbiota, including the suppression of the potential pathogen *g_Clostridium_T* and promotion of the beneficial bacterium *g_Lactococcus_A*, alongside increased concentrations of major short-chain fatty acids (acetate, propionate, and butyrate). These correlative observations suggest that EP may help mitigate the growth-inhibiting effects of SBM in *Monopterus albus*, offering a potential functional strategy for high-SBM aquafeeds.

## Simple Summary

The substitution of fish meal (FM) with soybean meal (SBM) is a sustainable strategy for the aquaculture industry, but it often leads to reduced feed intake and poor growth in farmed fish. This study aimed to evaluate whether adding earthworm powder (EP) to the diet could mitigate these negative impacts in rice field eels (*Monopterus albus*). Our findings indicate that while high levels of SBM suppressed appetite and damaged the gut, the inclusion of 2.5% EP effectively reversed these effects. EP was found to stimulate eels’ feeding behavior by regulating brain signals and hormones, while simultaneously repairing intestinal tissue, strengthening the gut barrier, and balancing the community of gut bacteria. These results suggest that EP is a promising, natural feed additive that can support the use of SBM proteins in aquafeeds, thereby promoting the health and sustainable farming of *Monopterus albus*.

## 1. Introduction

The limited availability, fluctuating supply, and rising costs of fish meal (FM) have emerged as significant constraints to the sustainable growth of the aquaculture industry [1]. Reducing FM inclusion levels while enhancing the utilization of plant protein sources is a primary strategy for cost reduction and resource conservation [2]. FM contains specific attractants that strongly stimulate the feeding behavior of fish, many of which utilize chemosensory systems to locate food [3]. This directly influences feeding rates and sustained intake—a notable deficiency in most plant-based proteins. For instance, research indicates that replacing a high proportion of FM with corn gluten meal significantly reduces the feeding rate of juvenile striped catfish (*Pangasianodon hypophthalmus*) [4]. Furthermore, the various antinutritional factors present in plant proteins can impair nutrient digestion, decrease feed palatability, and compromise intestinal integrity [2,5]. Consequently, identifying functional additives that effectively promote feeding and improve gut health has become essential to mitigate the adverse effects of high levels of plant protein inclusion.

In aquaculture, particularly during the larval rearing stage of carnivorous fish species, earthworms are widely used as a critical “starter feed” due to their high palatability and comprehensive nutritional profile, with considerable potential as feed attractants [6]. However, the high perishability and inconsistent supply of fresh earthworms have constrained their large-scale inclusion in formulated diets. Earthworms are detritivorous organisms capable of utilizing hard-to-process agro-forestry residues and biomass, such as straw, livestock manure, and pruning waste. Cultivating earthworms using such biomass and processing them into earthworm powder (EP) not only preserves their nutritional and flavor characteristics but also significantly improves storage stability and supply continuity, thus demonstrating considerable development potential. In terrestrial livestock research, EP has been shown to increase feed intake and modulate gut microbial homeostasis [7,8]. Similarly, preliminary aquatic studies indicate that EP supplementation can enhance nutrient digestibility and promote growth performance in fish [9]. Furthermore, studies on Nile tilapia (*Oreochromis niloticus*) have also found that replacing 25% of FM with EP significantly promoted fish growth and enhanced survival rates [10]. However, studies on rainbow trout (*Oncorhynchus mykiss*) have found that substituting fish meal with EP led to a significant decline in growth performance, yet it markedly promoted the activity of digestive enzymes [11].

The rice field eel (*Monopterus albus*, *M. albus*) is a prominent specialty aquaculture species in China, with an annual production exceeding 340,000 tons. In practical aquaculture production, it has been observed that fish rely heavily on olfactory and gustatory cues for feeding [12]. Consequently, feed palatability serves as a decisive factor for both the onset of feeding and subsequent growth performance. Current industry practices rely heavily on fresh earthworms or high-inclusion FM diets to ensure adequate attraction for *M. albus*; however, this reliance is increasingly unsustainable due to escalating costs. Exploring the application of EP in *M. albus* nutrition offers a promising pathway to alleviate FM dependency. Furthermore, EP supplementation may counteract the appetite suppression and intestinal health challenges induced by high-SBM diets, ultimately providing a sustainable feed solution for the low-fishmeal, sustainable cultivation of this species.

## 2. Materials and Methods

### 2.1. Feed Formulation

Experimental diets included a control (CON) formulated with fish meal, soybean meal, and chicken powder as dietary protein providers, alongside fish and soybean oils as lipid substrates. The nutritional profile was adjusted to align with the physiological needs of *M. albus*, targeting crude protein and lipid levels of 40% and 6%, respectively [13,14,15]. Two experimental variants were derived from this basal formulation: the SBM diet, where fish meal was reduced by 10% in exchange for a 14% increase in soybean meal; and the EP diet, which involved the addition of 2.5% earthworm powder (EP) to the SBM matrix (Table 1). The inclusion level of 2.5% EP was selected based on results from our preliminary pilot study, which indicated optimal palatability at this concentration.

Earthworm powder was purchased from Dazu Ecological Agriculture Co., Ltd., located in Nijiangkou Town, Heshan District, Yiyang City, Hunan Province, China. The purchased powder was prepared according to the supplier’s standardized process: fresh earthworms were rinsed, subjected to a brief starvation period for gut clearance, blanched, oven-dried at 60 °C, and then milled into a fine powder. No chemical additives, enzymes, or antioxidants were introduced during this manufacturing process. The proximate composition of the powder used in this experiment, as analyzed in our laboratory, was determined to contain 8.10% moisture, 60.71% crude protein, 6.04% crude fat, 12.17% crude ash, and 2.27% crude fiber (on a dry matter basis). Additionally, the free amino acid composition (in g/100g) of the earthworm powder was determined as follows: aspartic acid 5.09, threonine 2.52, serine 2.26, proline 1.98, glutamic acid 6.65, glycine 2.64, alanine 3.17, cystine 0.60, valine 2.92, methionine 0.86, isoleucine 2.49, leucine 4.29, tyrosine 1.63, phenylalanine 2.37, histidine 1.29, lysine 3.66, and arginine 3.53.

In terms of manufacturing, raw materials were ground to a fine powder, accurately aliquoted, and subjected to homogenization in a V-shell blender for a 5-min duration. Lipid components were incorporated during blending to enhance physical consistency. Post-preparation, the powdered feed underwent air-drying and was archived in a moisture-controlled, cool environment.

### 2.2. Feeding Management

The biological assay employed *M. albus* juveniles sourced from a commercial hatchery in Yiyang (Hunan, China). The study population comprised 360 eels (initial mass: 18.00 ± 0.01 g), which were allocated to nine floating nets (0.5 m × 0.5 m× 0.5 m) to establish three experimental groups (CON, SBM, EP) with three replicates per group (40 fish/replicate). Following a 7-day adaptation phase and a subsequent 24-h feed deprivation period, the 56-day feeding trial was initiated. Fish were counted and bulk-weighed at the start to ensure uniform initial biomass. Daily rations were administered at dusk (17:00–18:30) until visual satiety was observed, corresponding to approximately 3–5% of total biomass. Visual satiety was defined as the point at which fish showed no interest in feed for a period of 5 min. Throughout the trial, aquatic environmental indices were monitored and stabilized at: 28.6 ± 1.8 °C (temperature), 6.5 ± 0.4 mg/L (dissolved oxygen), 7.2 ± 0.3 (pH), and 0.23 ± 0.03 mg/L (ammonia nitrogen).

### 2.3. Sample Collection

Sampling protocols were executed in two phases. Fecal samples were collected under sterile conditions. For sampling, fish were anesthetized and sacrificed. The intestinal tract was aseptically dissected. The mid-intestine was then opened longitudinally using sterile scissors, and fecal contents were gently extruded using sterile forceps. Samples from two random fish per cage were immediately frozen in liquid nitrogen and then stored at −80 °C until analysis for gut microbiome (16S rRNA gene sequencing) and short-chain fatty acid (SCFA) quantification. Following this, the remaining stock underwent a 24-h feed deprivation period before census and weighing. For physiological assessments, six fish were extracted from each cage. Blood collected via the caudal vein from four individuals was processed into serum (incubation at 4 °C overnight; centrifugation at 3000 rpm, 10 min) and banked at −80 °C for hormonal profiling. Concurrently, whole brain and gut tissues were dissected. The mid-intestinal segment destined for histology was preserved in 4% paraformaldehyde, whereas the remaining tissue samples were aliquoted and stored at −80 °C for gene expression and biochemical quantification. All fish were anesthetized with MS-222 prior to dissection. For biochemical parameters and gene expression analyses, six fish were randomly sampled from each experimental group. For serum hormone measurements, samples from two individuals within the same cage were pooled prior to ELISA analysis due to the relatively high cost of the commercial assay kits, resulting in three pooled samples per group for statistical analysis.

### 2.4. Determination of Hydrolyzed Amino Acids

The amino acid profiles of the experimental diets and earthworm powder were determined using a Hitachi High-Tech automatic amino acid analyzer (Hitachi High-Tech Corporation, Tokyo, Japan). Samples were pre-treated via acid hydrolysis. Briefly, a precise amount of sample was mixed with 5 mL of HCl solution (1:1, *v*/*v*) and hydrolyzed at 110 °C for 22 h. After cooling to room temperature, the hydrolysate was transferred and diluted to a final volume of 10 mL, followed by filtration. A 0.05 mL aliquot of the filtrate was evaporated to dryness under a nitrogen stream. The resulting residue was reconstituted in 2 mL of 20 mM HCl, passed through a 0.22 μm membrane filter, and subjected to analysis.

### 2.5. Preparation of Intestinal Histological Sections

For histological analysis, mid-intestinal specimens underwent routine histological processing, including alcohol dehydration, xylene clearing, and embedding in paraffin blocks. To ensure the accuracy and consistency of villus height measurements, the mid-intestinal samples were meticulously oriented during the embedding process. Specifically, all samples were aligned to be sectioned transversely (perpendicular to the longitudinal axis of the intestine). Following sectioning, slides were stained with hematoxylin and eosin (H&E) to visualize tissue morphology. Digital imaging was conducted utilizing the Nikon Eclipse Ci-L microscopy platform (Nikon Corporation, Tokyo, Japan) and the KFBIO KF-FL-020 digital pathology scanner (Ningbo KF Bio-Tech Co., Ningbo, China). Villus height (VH) and Goblet cell (GC) were measured using ImageJ software (https://imagej.net/ij/, accessed on 9 March 2026).

### 2.6. Assay of Hormones in Serum

Serum hormone levels were quantified using ELISA kits. The panel of hormones assayed included Ghrelin (GHR, BP04948), Peptide YY (PYY, BP05023), Insulin (INS, BP05189), Leptin (LEP, BP05065), Cholecystokinin (CCK, BP05212), and Glucagon-like Peptide-1 (GLP-1, BP05059). Prior to the formal experiment, a pre-experiment was conducted using randomly selected samples for each indicator, and calibration curves were prepared (with a fit of >0.99). All commercial kits were obtained from Baipeng Biotech Co., Ltd. (Beijing, China). The assay procedures were conducted in strict accordance with the manufacturer’s protocols. For each replicate cage, one representative serum sample was processed, strictly adhering to the standard operating procedures provided by the supplier.

### 2.7. Measurement of Intestinal Biochemical Parameters

To determine intestinal biochemical indices, mid-intestinal specimens were mechanically homogenized in a 10-fold volume (1:9 dilution) of pre-chilled physiological saline under ice-bath conditions. Following centrifugation, the resulting supernatant was harvested.

Digestive capacity was assessed by measuring the activities of Amylase (AMS, C016-1-2), Lipase (LPS, A054-2-1), and Trypsin (TRY, A080-2-2). Antioxidant status and oxidative stress were evaluated via Catalase (CAT, A007-1-1), Superoxide Dismutase (SOD, A001-3-2), Glutathione (GSH, A006-2-1), Total Antioxidant Capacity (T-AOC, A015-2-1), Malondialdehyde (MDA, A003-1-2), and Hydrogen Peroxide (H_2_O_2_, A064-1-1) levels. All specific assays were conducted utilizing commercial colorimetric diagnostic kits supplied by Nanjing Jiancheng Bioengineering Institute (Nanjing, China).

### 2.8. Quantitative PCR Analysis

Total RNA was isolated from mid-intestine and brain tissues utilizing the Trizol reagent protocol. RNA quality was verified, showing an A260/A280 ratio ranging from 1.8 to 2.0, and agarose gel electrophoresis confirmed distinct bands with no signs of degradation. Subsequently, RNA was reverse-transcribed into cDNA. QPCR was performed using specific primers (Table 2), all of which were validated for specificity via melting curve analysis, ensuring amplification efficiencies between 90% and 110%. The relative expression levels of target genes were normalized to the reference gene *rpl-17* and calculated using the 2^−ΔΔCT^ method. In this study, the SBM group was used as the reference group for normalization in order to clearly evaluate both the inhibitory effects of the SBM diet and the potential mitigating effects of EP supplementation. Detailed thermal cycling programs and reaction systems are provided in the Supplementary File.

### 2.9. Intestinal 16S rRNA Sequencing and SCFA Analysis

Fecal specimens were harvested to facilitate dual analyses of the gut microbiota and SCFAs. The mid-intestine was opened longitudinally using sterile scissors, and fecal contents were gently extruded using sterile forceps.

Microbiome Profiling: Post-DNA extraction and quality control, the bacterial 16S rRNA gene (V3-V4 region) was targeted for PCR amplification. The resulting libraries underwent paired-end sequencing (2 × 300 bp) utilizing the Illumina MiSeq system (Illumina, Inc., San Diego, CA, USA). Bioinformatics analysis was conducted within the QIIME 2 (2020.6) framework. Specifically, the DADA2 pipeline was employed for primer removal, denoising, and sequence assembly to resolve Amplicon Sequence Variants (ASVs). To ensure data robustness, rare ASVs (<0.1% relative abundance) were excluded from the final taxonomic matrix.

SCFAs Analysis: The concentration of SCFAs was determined via a GC-MS approach. Briefly, samples were acidified with phosphoric acid and homogenized. Extraction was performed twice using diethyl ether in an ice bath. The resulting organic layers were combined and adjusted to a fixed volume prior to injection into a Trace1310-ISQ7000 GC-MS analyzer (Thermo Fisher Scientific, Waltham, MA, USA).

### 2.10. Data Processing and Analysis

Statistical analyses were conducted using SPSS software (v26.0), while data visualization was performed using Python (V3.9). The net cage was considered the experimental unit for statistical analyses, and sampled individuals were used to obtain representative biological measurements for each replicate. The normality of the data distribution was assessed via the Shapiro–Wilk test, and homogeneity of variance was verified using Bartlett’s test. For datasets satisfying both assumptions, a one-way analysis of variance was employed, followed by Tukey’s HSD post hoc test to identify significant differences between groups. Conversely, data that did not meet the criteria for normality or homogeneity were analyzed using the Kruskal–Wallis test. In cases where significant differences were detected, Dunn’s test was applied for pairwise comparisons. All statistical tests were considered significant at *p* < 0.05.

## 3. Results

### 3.1. Appetite Analysis in the Organism

#### 3.1.1. Growth Performance and Feeding Rate

To evaluate the impact of dietary treatments on *M. albus*, growth and feeding parameters were analyzed. As shown in Table 3, no significant differences in SR were observed among the groups (*p* > 0.05). However, compared to the CON group, the SBM group exhibited a significant reduction in both WGR, SGR, and DFI, along with a marked increase in FCR (*p* < 0.05). Notably, the EP group significantly ameliorated these declines, improving both WGR, SGR, and DFI (*p* < 0.05), although its WGR and SGR remained numerically lower and its FCR markedly higher than those of the CON group (*p* < 0.05).

#### 3.1.2. Amino Acid Composition

**Figure 1:**
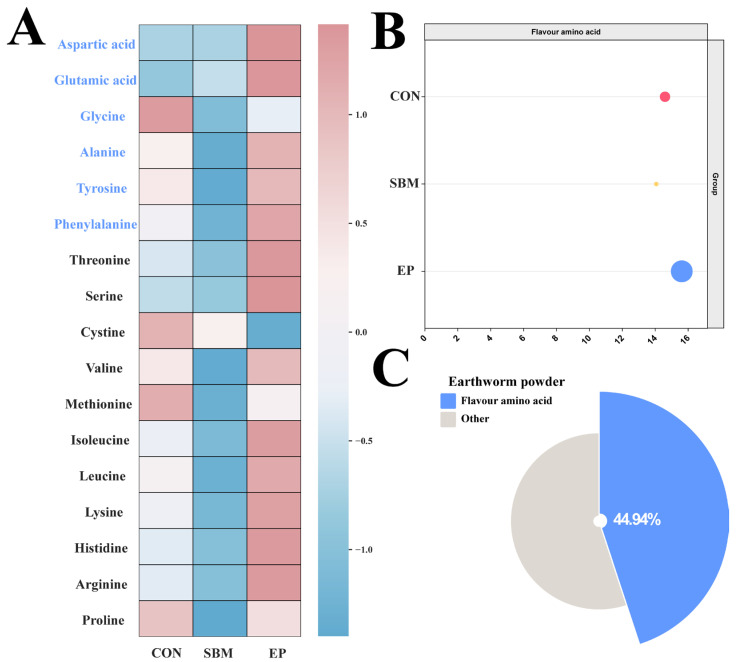
Amino Acid Composition. (**A**) Heatmap illustrating the standardized concentration (z-score) and distribution of amino acids across groups, where color intensity represents standardized abundance levels; (**B**) Lollipop plot illustrating the total content of flavor amino acids across experimental diets. The circle size represents the absolute content (g/100g), and the x-axis denotes the total flavor amino acid content (g/100g); (**C**) Pie Chart of flavor amino acid content in the Earthworm powder. Flavor amino acids: a category of amino acids that contribute specific palatable or umami tastes, primarily including glutamic acid, aspartic acid, phenylalanine, alanine, glycine, and tyrosine.

Analysis of the dietary amino acid profiles revealed that the EP diet contained higher levels of aspartic acid, glutamic acid, alanine, tyrosine, and phenylalanine compared to the CON and SBM groups (Figure 1A), although the CON group exhibited the highest glycine content. Furthermore, the total content of flavor amino acids in the EP diet was superior to that of the CON and SBM groups (Figure 1B). Further analysis of the earthworm powder indicated that flavor amino acids accounted for 44.94% of its total amino acid composition (Figure 1C).

#### 3.1.3. Molecular and Physiological Mechanisms of Appetite Regulation

**Figure 2:**
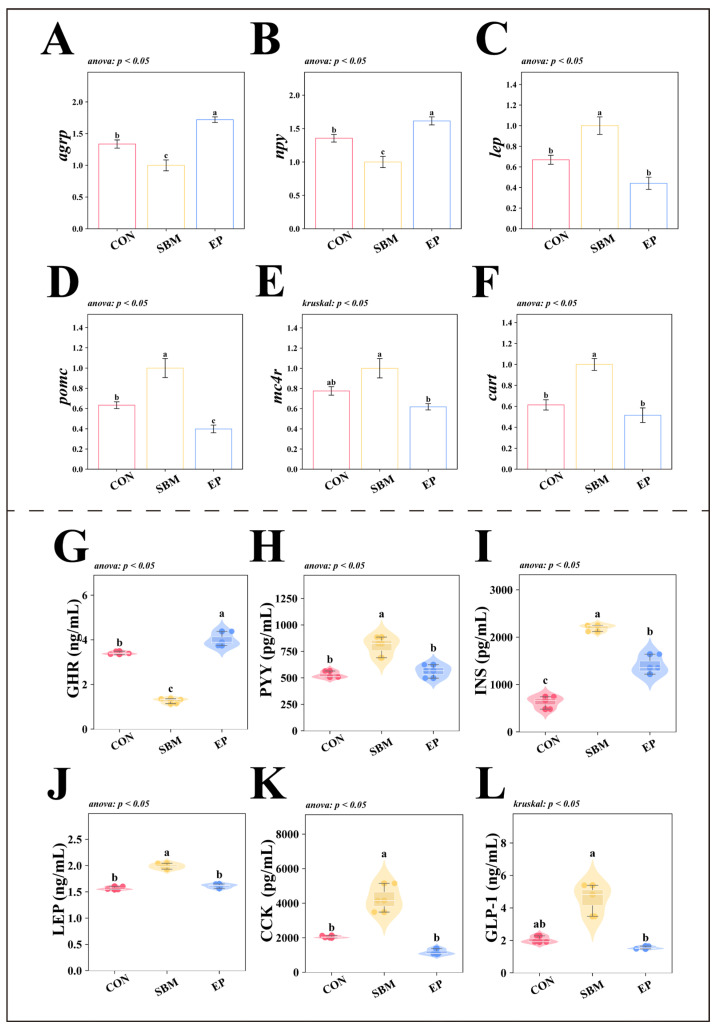
Molecular and Physiological Mechanisms of Appetite Regulation. Values are presented as mean ± SEM. Individual data points represent biological observations. For gene expression analyses (**A**–**F**), *n* = 6 biological samples per group. For serum hormone measurements (**G**–**L**), *n* = 3 pooled samples per group; Different superscript letters indicate significant differences among groups (*p* < 0.05), determined by one-way ANOVA followed by Tukey’s HSD test or Kruskal–Wallis test followed by Dunn’s test, as indicated in the respective panels; (**A**) *agrp*: *agouti-related protein*; (**B**) *npy*: *neuropeptide y*; (**C**) *lep*: *leptin*; (**D**) *pomc*: *pro-opiomelanocortin*; (**E**) *mc4r*: *melanocortin 4 receptor*; (**F**) *cart*: *cocaine and amphetamine-regulated transcript*; (**G**) GHR (ng/mL): Ghrelin; (**H**) PYY (pg/mL): Peptide YY; (**I**) INS (pg/mL): Insulin; (**J**) LEP (ng/mL): Leptin; (**K**) CCK (pg/mL): Cholecystokinin; (**L**) GLP-1 (ng/mL): Glucagon-Like Peptide-1. (**G**–**L**) Violin plots, box plots, scatter points, and swarm plots are combined to visualize the data distribution. The swarm arrangement slightly offsets points horizontally to avoid overlap and improve visualization of individual observations.

At the molecular level, the expression of orexigenic genes (*agrp*, *npy*) was significantly downregulated in the SBM group (*p* < 0.05; Figure 2A,B), while anorexigenic genes (*lep*, *pomc*, *mc4r*, *cart*) were significantly upregulated (*p* < 0.05; Figure 2C–F). The EP group successfully reversed this trend, restoring the expression profiles of these appetite-regulating genes.

Moreover, the SBM group showed significantly lower serum GHR levels (*p* < 0.05; Figure 2G), alongside elevated concentrations of satiety hormones, including PYY, INS, LEP, CCK, and GLP-1 (*p* < 0.05, Figure 2H–L). The EP group significantly increased ghrelin levels and reduced the concentrations of these anorexigenic hormones (*p* < 0.05).

### 3.2. Analysis of Intestinal Tissue Health

#### 3.2.1. Intestinal Histomorphology and Digestive Capacity

**Figure 3:**
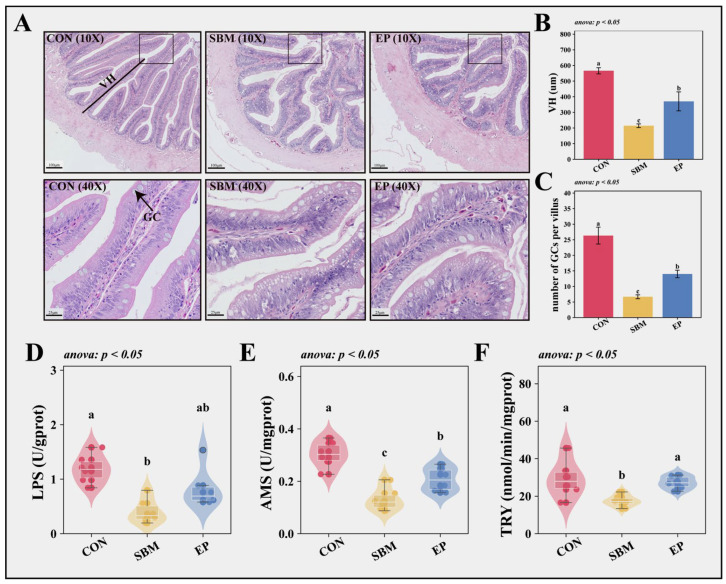
Intestinal Histomorphology and Digestive Capacity. Values are presented as mean ± SEM (*n* = 6); Different superscript letters indicate significant differences among groups (*p* < 0.05), determined by one-way ANOVA followed by Tukey’s HSD test or Kruskal–Wallis test followed by Dunn’s test, as indicated in the respective panels; (**A**) HE staining of intestine; (**B**) VH (um): Villus Height; (**C**) GC (unit/root): Goblet Cell; (**D**) LPS (U/gprot): Lipase; (**E**) AMS (U/mgprot): Amylase; (**F**) TRY (nmol/min/mgprot): Trypsin. (**D**–**F**) Violin plots, box plots, scatter points, and swarm plots are combined to visualize the data distribution. The swarm arrangement slightly offsets points horizontally to avoid overlap and improve visualization of individual observations.

Histological analysis using H&E staining (Figure 3A) revealed that dietary SBM inclusion significantly compromised intestinal integrity. Compared to the CON group, the SBM group exhibited villus atrophy, indicated by significantly reduced VH, and a marked depletion in GC abundance (*p* < 0.05; Figure 3B,C). Concomitantly, the activities of digestive enzymes (LPS, AMS, and TRY) were significantly depressed (*p* < 0.05; Figure 3D–F). However, the EP group effectively alleviated these defects, restoring VH, GC counts, and digestive enzyme activities (*p* < 0.05).

#### 3.2.2. Oxidative Stress and Antioxidant Defense Mechanisms

**Figure 4:**
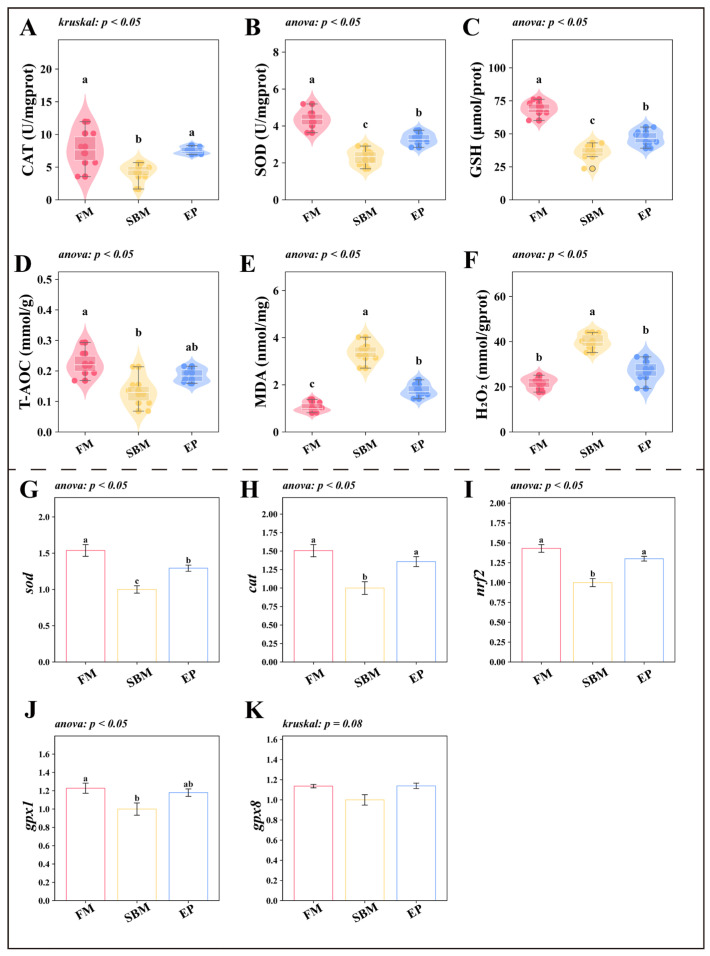
Oxidative Stress and Antioxidant Properties. Values are presented as mean ± SEM (*n* = 6); Different superscript letters indicate significant differences among groups (*p* < 0.05, determined by One-way ANOVA followed by Tukey’s HSD test or Kruskal–Wallis followed by Dunn’s test, as indicated in the respective panels); (**A**) CAT (U/mgprot): Catalase; (**B**) SOD (U/mgprot): Superoxide Dismutase; (**C**) GSH (umol/prot): Glutathione; (**D**) T-AOC (mmol/g): Total Antioxidant Capacity; (**E**) MDA (nmol/mg): Malondialdehyde; (**F**) H_2_O_2_ (mmol/gprot): Hydrogen Peroxide; (**G**) *sod*: *superoxide dismutase*; (**H**) *cat*: *catalase*; (**I**) *nrf2*: *nuclear factor erythroid 2-related factor*; (**J**) *gpx1*: *glutathione peroxidase* 1; (**K**) *gpx8*: *glutathione peroxidase 8*. (**A**–**F**) Violin plots, box plots, scatter points, and swarm plots are combined to visualize the data distribution. The swarm arrangement slightly offsets points horizontally to avoid overlap and improve visualization of individual observations.

The SBM diet induced a state of oxidative stress in the intestine. Biochemical assays showed a significant reduction in antioxidant parameters (CAT, SOD, GSH, and T-AOC) alongside a surge in oxidative damage markers (MDA and H_2_O_2_) in the SBM group (*p* < 0.05; Figure 4A–F). These changes were mirrored at the transcriptional level: the expression of antioxidant defense genes (*sod*, *cat*, *gpx1*) and the master regulator *nrf2* was significantly downregulated (*p* < 0.05; Figure 4G–K). Conversely, the EP group significantly boosted the antioxidant system, upregulating *nrf2* and downstream antioxidant genes, and reducing MDA and H_2_O_2_ accumulation (*p* < 0.05).

#### 3.2.3. Intestinal Barrier Function and Inflammatory Responses

**Figure 5:**
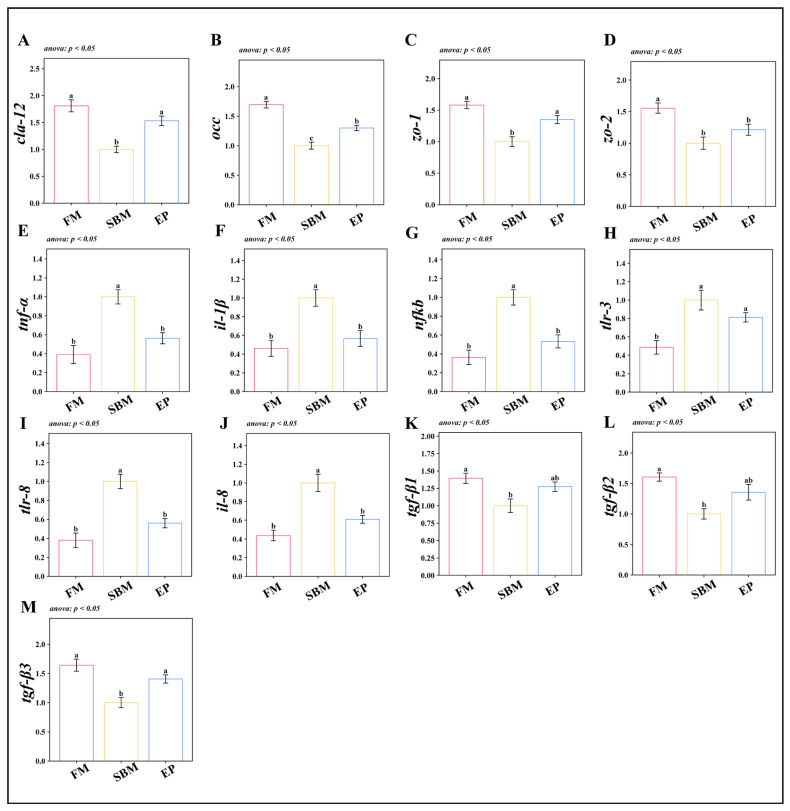
Intestinal Barrier Function and Inflammatory Responses. Values are presented as mean ± SEM (*n* = 6); Different superscript letters indicate significant differences among groups (*p* < 0.05, determined by One-way ANOVA followed by Tukey’s HSD test or Kruskal–Wallis followed by Dunn’s test, as indicated in the respective panels); (**A**) *cla-12*: *claudin-12*; (**B**) *occ*: *occludin*; (**C**) *zo-1*: zonula occludens-1; (**D**) *zo-2*: *zonula occludens-2*; (**E**) *tnf-α*: *tumor necrosis factor-alpha*; (**F**) *il-1β*: interleukin-1 beta; (**G**) *nfkb*: *nuclear factor kappa b*; (**H**) *tlr-3*: *toll-like receptor 3*; (**I**) *tlr-8*: *toll-like receptor 8*; (**J**) *il-8*: *interleukin-8*; (**K**) *tgf-β1*: *transforming growth factor-beta 1*; (**L**) *tgf-β2*: *transforming growth factor-beta 2*; (**M**) *tgf-β3*: *transforming growth factor-beta 3*.

Gene expression analysis highlighted a disruption in mucosal barrier function. In the SBM group, genes encoding tight junction proteins (*cla-12*, *occ*, *zo-1*, *zo-2*) and anti-inflammatory cytokines (*tgf-β1*, *tgf-β2*, *tgf-β3*) were significantly downregulated (*p* < 0.05, Figure 5A–D and 5K–M). Conversely, pro-inflammatory markers (*tnf-α*, *il-1β*, *nfkb*, *tlr-3*, *tlr-8*, *il-8*) were significantly upregulated (*p* < 0.05, Figure 5E–J). The EP group reversed this inflammatory profile, significantly upregulating physical barrier genes (except *zo-2*) and *tgf-β3*, while suppressing the expression of key pro-inflammatory mediators (*p* < 0.05).

### 3.3. Gut Microbiota and SCFAs

#### 3.3.1. Microbial Diversity and Community Structure

**Figure 6:**
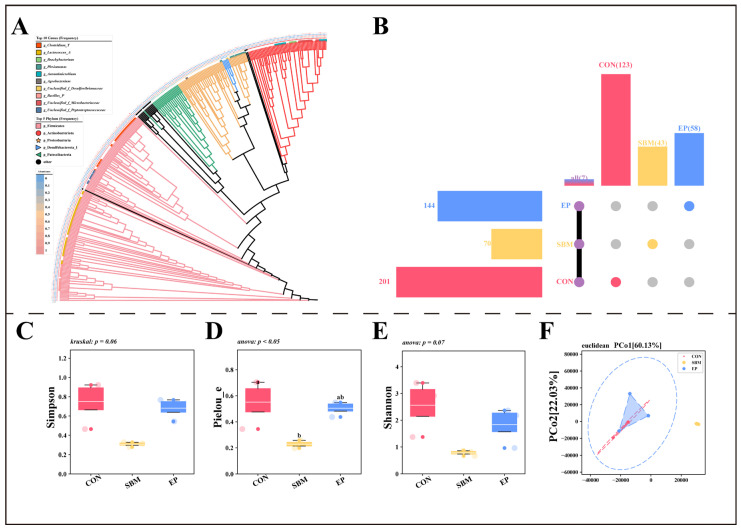
Microbial Diversity and Community Structure. Values are presented as mean ± SEM (*n* = 3). Different superscript letters indicate significant differences among groups (*p* < 0.05, determined by One-way ANOVA followed by Tukey’s HSD test or Kruskal–Wallis followed by Dunn’s test, as indicated in the respective panels); (**A**) Phylogenetic tree constructed based on ASV feature sequences; (**B**) The upset plot shows the number of ASVs in each group; (**C**–**E**) Alpha diversity indices; (**F**) Beta diversity based on Euclidean distance.

According to the phylogenetic tree based on ASV sequences (Figure 6A), p_Firmicutes, p_Actinobacteriota, and p_Proteobacteria were the three most common phyla in all groups. At the genus level, *g_Clostridium_T*, *g_Lactococcus_A*, and *g_Brachybacterium* were the dominant taxa. Regarding ASV composition (Figure 6B), the CON, SBM, and EP groups contained 201, 70, and 144 ASVs, respectively. The number of unique ASVs was 123 in the CON group, 43 in the SBM group, and 58 in the EP group, with only 7 ASVs shared by all three groups.

In terms of Alpha diversity, the SBM group showed lower Simpson and Shannon indices compared to the CON group, and the Pielou_e index decreased significantly (*p* < 0.05, Figure 6C–E). However, these diversity indicators increased in the EP group. PCA (Beta diversity) based on Euclidean distance (Figure 6F) further indicated that the microbial structure of the SBM group was clearly different from the CON group, while the EP group showed a trend of moving back toward the CON group.

#### 3.3.2. Changes in Microbial Composition

**Figure 7:**
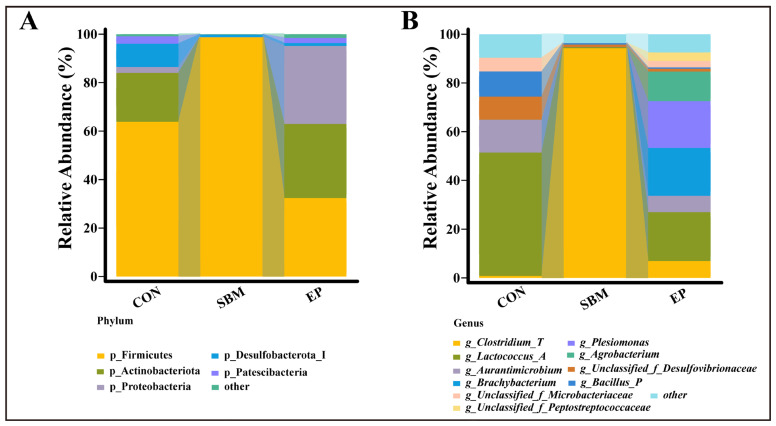
Changes in Microbial Composition. (**A**) The phylum composition of gut microbiota in each group (Top 5); (**B**) The genus composition of gut microbiota in each group (Top 10).

At the phylum level (Figure 7A), the SBM diet increased the abundance of p_Firmicutes and decreased p_Proteobacteria compared to the CON group. This trend was reversed in the EP group, where p_Firmicutes decreased, and p_Proteobacteria increased. At the genus level (Figure 7B), *g_Clostridium_T* abundance increased while *g_Lactococcus_A* decreased in the SBM group. After the EP intervention, these changes were reversed.

#### 3.3.3. Fecal SCFA Levels and Correlation Analysis

**Figure 8:**
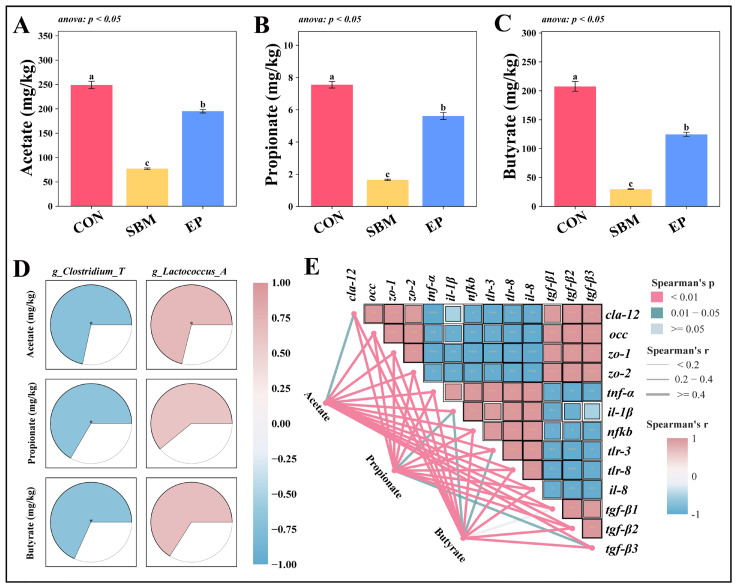
Fecal SCFA Levels and Correlation Analysis. Values are presented as mean ± SEM (*n* = 3). Different superscript letters indicate significant differences among groups (*p* < 0.05, determined by One-way ANOVA followed by Tukey’s HSD test or Kruskal–Wallis followed by Dunn’s test, as indicated in the respective panels); (**A**–**C**) Fecal SCFA levels (Acetate, Propionate, and Butyrate); (**D**) Correlation analysis between intestinal microbiota and SCFAs; (**E**) Correlation analysis between SCFAs and intestinal barrier function. In a correlation heatmap, the asterisks (*, **, ***) represent the statistical significance levels, typically indicating *p* < 0.05, *p* < 0.01, and *p* < 0.001, respectively.

The levels of fecal SCFAs showed that acetate, propionate, and butyrate were significantly lower in the SBM group than in the CON group (*p* < 0.05, Figure 8A–C). After adding EP, the concentrations of these three SCFAs increased significantly (*p* < 0.05). Correlation analysis (Figure 8D) found that *g_Clostridium_T* was negatively correlated with acetate, propionate, and butyrate, while *g_Lactococcus_A* was positively correlated with acetate. Additionally, these SCFAs were significantly associated with the expression of several genes related to intestinal barrier function (Figure 8E).

## 4. Discussion

### 4.1. EP Enhances Growth in M. albus by Promoting Feeding Activity

Elevated growth performance is intrinsically linked to higher feed consumption, which is primarily governed by the palatability of the diet [16]. In line with previous research [17,18], replacing a substantial portion of FM with SBM significantly suppressed the appetite of *M. albus*. However, dietary inclusion of EP effectively countered this negative effect and bolstered ingestion, acting as the driver for improved growth metrics.

Regarding chemosensory pathways, amino acids serve as natural attractants that trigger specific olfactory and gustatory responses in teleosts [19,20]. It has been documented that flavor amino acids, particularly alanine, act as efficient gustatory stimulants for various fish [21]. Our analysis revealed that EP is abundant in these essential flavor compounds, effectively replenishing the flavor profile diminished by SBM inclusion. Consequently, EP enhanced the diet’s attractability via chemosensory signaling, which in turn stimulated the feeding activity of *M. albus*.

The neuroendocrine system eventually integrates and processes these chemical signals. Dietary cues are relayed to the feeding center via the “olfactory-brain axis,” where they orchestrate the expression of genes and hormones involved in appetite control [22,23]. This experiment found that adding EP to the diet elevated the transcription of orexigenic factors and suppressed anorexigenic hormone levels. These findings imply that EP mitigates the regulatory disruptions caused by SBM-based diets, thus promoting appetite and consumption from a mechanistic perspective. However, the precise upstream pathway linking EP flavor compounds to brain gene expression remains to be fully elucidated.

It is important to note that while DFI in the EP group was higher compared to CON, the growth performance was improved but still lower than CON, and FCR was not effectively reduced. This suggests that the primary growth-promoting effect of EP in this context is likely driven by enhanced feed attractability and increased intake, and the resulting excessive feed intake may surpass the organism’s actual requirements, leading to nutrient waste and subsequently increasing FCR. Furthermore, the observed changes in appetite-related gene expression and hormone levels may be secondary to this increased feed intake rather than a direct regulatory effect of EP.

### 4.2. EP Facilitates Growth in M. albus by Optimizing Intestinal Digestion and Absorption

Beyond ensuring adequate ingestion, preserving intestinal health is fundamental to maximizing nutrient assimilation efficiency. The adverse effects of antinutritional factors in SBM on the fish gut have been well-documented [24,25]. Our results indicate that dietary EP inclusion successfully mitigated SBM-triggered intestinal lesions and boosted digestive enzyme activities. This constitutes another major intrinsic factor for the growth-promoting effect of EP.

Mechanistically, this study demonstrates that EP attenuates SBM-induced oxidative stress in the gut, preserves mucosal barrier integrity, and suppresses inflammatory processes. The intestinal antioxidant system serves as a primary defense for enterocyte health, which is critically linked to the overall robustness of the gut barrier [26]. A functional barrier acts as a shield against deleterious agents, subsequently minimizing localized inflammation [27]. By safeguarding intestinal homeostasis via these pathways, EP indirectly fosters nutrient uptake and organismal development.

These protective benefits are likely attributable to the specific bioactive compounds within EP. Specifically, lumbrokinase, a well-characterized fibrinolytic enzyme complex naturally found in earthworms, is reported to possess not only thrombolytic activity but also anti-inflammatory [28]. It has been shown to down-regulate *nfkb* expression through activation of the *sirt1* pathway, thereby dampening oxidative stress and inflammatory cascades at the molecular level [29]. While our data indicate an association between EP supplementation and enhanced *nrf2*-mediated antioxidant capacity, the direct interaction between specific EP-derived bioactive peptides (e.g., lumbrokinase) and the *nrf2* pathway remains to be fully elucidated. Future studies employing techniques such as chromatin immunoprecipitation-qPCR (ChIP-qPCR) would be valuable to directly verify the binding of activated *nrf2* to antioxidant response elements (AREs) in the promoters of its target genes (e.g., *cat*, *sod*), thereby providing direct evidence for this proposed mechanism. Additionally, the high concentration of amino acids, such as glutamate, provides essential energy substrates and precursors for enterocytes. This directly facilitates mucosal regeneration and barrier reinforcement [30], effectively offsetting the nutritional deficiencies or imbalances characteristic of SBM-based diets. Since there was no direct quantitative measurement of specific bioactive compounds (e.g., lumbrokinase) in our experimental diets, these molecular mechanisms remain speculative and require careful interpretation pending future quantitative studies.

### 4.3. EP Modulates Gut Microbiota in M. albus

Beyond maintaining intestinal structure and function, the gut microbial ecosystem plays a pivotal role in nutrient metabolism and host health maintenance [31]. Microbial diversity serves as a critical indicator of taxonomic stability and functional robustness [32]. Our findings indicated that the high-level replacement of FM with SBM significantly reduced intestinal Alpha diversity, a trend consistent with various fish studies [33], suggesting a weakened microbial stability. This observed association suggests a potential link between EP and the maintenance or remodeling of the gut microbial architecture.

Regarding microbial composition, high-SBM inclusion significantly increased the abundance of *g_Clostridium_T*. This genus encompasses several known pathogens capable of producing exotoxins that directly compromise intestinal tight junctions, elevate mucosal permeability, and aggressively recruit inflammatory cells, thereby triggering or exacerbating enteritis [34,35]. Conversely, dietary EP treatment promoted the enrichment of *g_Lactococcus_A* while simultaneously curtailing the growth of *g_Clostridium_T*. Existing literature identifies *g_Lactococcus_A* as a key producer of SCFAs [36,37]. Although this study detected a significant positive correlation between *g_Lactococcus_A* and acetate levels, this genus possesses the metabolic potential to produce various SCFAs. Thus, its increased abundance likely synergistically promoted the recovery of overall SCFA levels. The observed negative correlation between EP and the abundance of *g_Clostridium_T* could be related to the presence of antimicrobial peptides (AMPs) in earthworms. Previous studies indicate that AMPs can specifically inhibit pathogen growth and regulate gut microbiota [38]. However, lacking direct quantification of AMPs in our diet, this remains a speculative hypothesis.

As core functional outputs of the gut microbiota, SCFAs are essential for maintaining the energy supply to intestinal epithelia, reinforcing barrier function, and regulating immune responses [39,40]. This study confirmed that high-SBM diets significantly depleted the concentrations of major SCFAs, including acetate, propionate, and butyrate, while EP intervention effectively reversed this decline. Correlation analysis further revealed that fluctuations in SCFA levels were closely linked to the abundances of *g_Clostridium_T* (significant negative correlation) and *g_Lactococcus_A* (significant positive correlation). Collectively, these interlinked correlations outline a potential associative pathway. The dietary inclusion of EP is associated with an increased abundance of beneficial bacteria like *g_Lactococcus_A*. This shift may favor the synthesis and secretion of SCFAs. SCFAs are known to directly nourish the intestinal epithelium and fortify the barrier [41]. Furthermore, they might contribute to an environment that limits opportunistic pathogens like *g_Clostridium_T*, possibly through lowering luminal pH and producing antimicrobial substances [42]. This associative restoration of microbial equilibrium supports intestinal health at the micro-ecological level. It works in synergy with the structural and functional improvements discussed earlier to facilitate overall growth performance. It is important to note that these relationships, while strongly correlated, are observational. Future studies employing functional approaches, such as microbiota transplantation, are needed to establish direct causality.

### 4.4. Considerations Regarding Sampling Strategy

In the present study, each net cage initially contained 40 fish, whereas not all individuals were randomly sampled from each cage for physiological and biochemical analyses. The relatively higher stocking number was intentionally adopted as a precautionary measure to minimize the risk of experimental failure due to potential mortality during the long-term culture period, which has occasionally occurred in previous eel culture trials. Random sampling of a limited number of individuals from each experimental unit is a commonly applied strategy in aquaculture nutrition studies when performing intensive biochemical and molecular analyses. In addition, the number of analyzed individuals was partly constrained by the high cost associated with multiple biochemical, gene expression, and microbiome analyses.

## 5. Conclusions

In summary, the present study demonstrates that dietary EP supplementation is associated with the alleviation of growth retardation induced by high-SBM diets in *M. albus*. Although the FM-based control diet (CON) resulted in the highest numerical growth and lowest FCR, the inclusion of EP correlated with multiple beneficial effects. These observations suggest a dual mechanism: EP may enhance dietary palatability via its flavor-active compounds, supporting increased feeding motivation and correlating with the modulation of appetite-related neuroendocrine pathways. Concurrently, EP supplementation was linked to improvements in intestinal structure and function, as well as favorable shifts in gut microbiota composition and short-chain fatty acid profiles. Collectively, these associative findings indicate that while challenges remain in fully replacing FM, EP shows promise as a functional ingredient for supporting sustainable aquafeed development for *M. albus*. Future studies are warranted to establish direct causal relationships.

## Figures and Tables

**Table 1:** Composition and nutrient level of experimental diet (%, dry matter).

Ingredients	CON	SBM	EP
^a^ Fish meal	42.00	32.00	32.00
^b^ Soybean meal	18.00	32.00	32.00
Chicken powder	4.00	4.00	2.00
Beer yeast	5.00	5.00	5.00
Fish oil	2.00	2.70	2.75
Soybean oil	0.20	0.00	0.00
^c^ Microcrystalline cellulose	5.60	1.10	0.55
α-starch	19.66	19.66	19.66
Choline	0.50	0.50	0.50
Ca(H_2_PO_4_)_2_·H_2_O_2_	2.00	2.00	2.00
^d^ Premix	1.00	1.00	1.00
^e^ Antioxidants	0.01	0.01	0.01
^f^ Mold inhibitor	0.03	0.03	0.03
Earthworm powder (EP)	0.00	0.00	2.50
^g^ Proximate composition (%)			
^h^ Crude protein	41.25	40.99	40.975
^i^ Crude lipid	6.42	6.40	6.40

Note: ^a^ The fish meal used was steam-dried Peruvian fish meal. ^b^ The soybean meal was standard commercial dehulled, defatted soybean meal without additional thermal autoclaving, maintaining typical residual antinutritional factor profiles. ^c^ Microcrystalline cellulose was used as an inert filler to adjust for differences in ingredient inclusion and to ensure all diets were iso-nitrogenous and iso-lipid. ^d^ Provided by MGO Ter Bio-Tech Co., Ltd. (Qingdao, China). Vitamin and Mineral Premix composition (mg/kg diet): KI (1%) 100 mg, CoCl_2_·6H_2_O (1%) 50 mg, CuSO_4_·5H_2_O 4 mg, FeSO_4_·H_2_O 120 mg, ZnSO_4_·H_2_O 60 mg, MnSO_4_·H_2_O 150 mg, Na_2_SeO_3_·5H_2_O (1%) 10 mg, MgSO_4_·H_2_O 30 mg, VB_1_ 5 mg, riboflavin 8 mg, VB_6_ 6 mg, VB_12_ 0.02 mg, VK_3_ 5 mg, inositol 100 mg, pantothenic acid 20 mg, niacin acid 30 mg, folic acid 1.7 mg, biotin 0.05 mg, VA 15 mg, VD_3_ 0.375 mg, VE 40 mg, VC 100 mg. ^e^ The main component of antioxidant is ethoxyquin. ^f^ The main component of the mold inhibitor is calcium propionate. ^g^ The proximate composition was measured. ^h^ Crude protein was measured using the Kjeldahl method. ^i^ Crude lipid was determined by the Soxhlet extraction method.

**Table 2:** Real-time PCR primer sequences.

Gene	Accession No.	Forward Primer (5′-3′)	Reverse Primer (5′-3′)
*rpl-17*	XM_020587712.1	CGAGAACCCGACTAAATCA	GTTGTAGCGACGGAAAGG
*agrp*	XM_020602674.1	TCTGCTATTGCCGCAGAGTT	TGTTTCTGGCTCCCCATCTC
*npy*	XM_020605952.1	TCCGTCTGAACCTCTGGGAA	CCGGGGTTCTCCGGTTTTAC
*lep*	XM_020613040.1	AGTGCACATCCCAGGAAATACT	AGCTGTTCAGCCATCCACTT
*pomc*	HQ864319.1	GAAACCCGTCGGTCGAAAAC	GGGGAAAACTTCAGCGGACT
*mc4r*	MF085052.1	CCCGTCACGCTGATTAGAAG	ATGGTGCAGGAGGTCCAGAT
*cart*	XM_020589950.1	GAACCCTGCGGGATTTCTAC	TCCGTGCGCCTTTCCTTAT
*sod*	XM_020598412.1	GTTGCCAAGATAGACATCACGG	TCATTGCCTCCTTTTCCCAG
*cat*	XM_020624985.1	CATTGGGAAGACTACACCTATCGC	GATGAAGAAGATGGGGGTGTTG
*nrf2*	XM_020596408.1	TCACAGACGAGAATGATGCC	CTGCTACTGGGAACTGAAACTG
*gpx1*	XM_020607739.1	AGATGTGAATGGGAAGGATGCC	AAACTTCGGGTCAGTCATCAGG
*gpx8*	XM_020593975.1	ATCCTGCCTTCAGATTCCTCAC	TCATTTCTCGCACCAGCACT
*cla-12*	XM_020607277.1	TCGTCACCTTCAATCGCAAC	CAGCCATGCACAGCAGTAAAG
*occ*	XM_020616177.1	GAGGCATTAGCATTAGTGTTGGG	AAATCTTGCTCCGGGTCTTGTAG
*zo-1*	XM_020621576.1	TGCGTTCCAACCACTATGACC	TACGGGTAGGATAACTGAGGTGG
*zo-2*	XM_020615114.1	TCAGCCATGAGTGCAGATTACC	CTCTTTGCTCCCTGACCTTCTC
*tnfα*	XM_020624826.1	CCAGGACACGATGGAGACAGTT	GAGGTTTTTATGGCTGGGTTGT
*il-1β*	KM262825.1	GTCAACCTCATTATCGCCACG	AAACTCCTCTTCTGGCTGTCG
*nfkb*	KC841853.1	CTTCGTAACCCAGAGGATAAACC	CAGATAAACACTGCACAGCCAAG
*tlr-3*	NW_018128024.1	CACCGCTCTGAAGAAAGATGAC	TCAAAACAACCAGGCTCCAG
*tlr-8*	XM_020596483.1	TTTGTCCTAACAGAGGGCTACG	CAGCATCAGCAGCACAATCAC
*il-8*	XM_020597092.1	CAACTCCCACTGCAAAGATACTG	CGACTTTGCCAGTTTCCTTTC
*tgf-β1*	XM_020605575.1	AAGTCCAGCAAGCAATCCCTAG	GAGATGCTTGTTGGGTCCTTG
*tgf-β2*	XM_020622328.1	AGCGAAGCGAGGAGGAGTATTAC	CAGCCTTCACCAAGTTAGATGC
*tgf-β3*	XM_020590885.1	GCCAAGAAAAACGAACAGAGG	TGTCACATCAAAGGAGACCCAC

Note: *rpl-17*: ribosomal protein L17; *agrp*: agouti-related peptide; *npy*: neuropeptide Y; *lep*: leptin; *pomc*: pro-opiomelanocortin; *mc4r*: melanocortin-4 receptor; *cart*: cocaine- and amphetamine-regulated transcript; *sod*: superoxide dismutase; *cat*: catalase; *nrf2*: nuclear factor erythroid 2–related factor 2; *gpx1*: glutathione peroxidase 1; *gpx8*: glutathione peroxidase 8; *cla-12*: claudin-12; *occ*: occludin; *zo-1*: zonula occludens-1; *zo-2*: zonula occludens-2; *tnfα*: tumor necrosis factor alpha; *il-1β*: interleukin-1 beta; *nfkb*: nuclear factor kappa b; *tlr-3*: toll-like receptor 3; *tlr-8*: toll-like receptor 8; *il-8*: interleukin-8; *tgf-β1*: transforming growth factor beta 1; *tgf-β2*: transforming growth factor beta 2; *tgf-β3*: transforming growth factor beta 3.

**Table 3:** Growth Performance.

Item	FM	SBM	EP
IBW (g)	18.27 ± 0.04	18.0 ± 0.10	18.03 ± 0.07
FBW (g)	42.18 ± 0.01 ^a^	30.34 ± 0.54 ^c^	36.66 ± 0.42 ^b^
SR (%) ^1^	85.00 ± 8.66	85.00 ± 8.66	83.33 ± 1.92
WGR (%) ^2^	130.48 ± 0.28 ^a^	68.54 ± 2.12 ^c^	103.3 ± 3.06 ^b^
DFI (%/d) ^3^	1.78 ± 0.01 ^b^	1.46 ± 0.08 ^c^	2.04 ± 0.04 ^a^
SGR (%/d) ^4^	1.49 ± 0.01 ^a^	0.93 ± 0.02 ^c^	1.27 ± 0.03 ^b^
FCR ^5^	1.22 ± 0.01 ^b^	1.55 ± 0.12 ^a^	1.62 ± 0.02 ^a^

Note: Values are presented as mean ± SEM (*n* = 3); Different superscript letters indicate significant differences among groups (*p* < 0.05), determined by one-way ANOVA followed by Tukey’s HSD test or Kruskal–Wallis test followed by Dunn’s test, as indicated in the respective panels; ^1^: SR (%): Survival rate = 100* Final number/Initial number; ^2^: WGR (%): Weight gain rate = 100 × (Final body weight − Initial body weight)/Initial body weight; ^3^: DFI (%/d): Daily feed intake = Total feed intake/[Experimental period × (Final body weight + Initial body weight)/2] × 100; ^4^: SGR (%/d): Specific growth rate = 100 × [ln(Final body weight) − ln(Initial body weight)]/days; ^5^: FCR: Feed conversion ratio = feed weight/(Final body weight − Initial body weight).

## Data Availability

The datasets generated during and/or analyzed during the current study are available from the corresponding author on reasonable request. Additionally, the raw 16S rRNA gene amplicon sequencing datasets have been deposited in the NCBI Sequence Read Archive (SRA) database under the BioProject accession number PRJNA1430104.

## Supplementary Materials

The following supporting information can be downloaded at: https://www.mdpi.com/article/10.3390/biology15060456/s1. All raw metadata generated during this study, including qPCR Ct values and histology scoring sheets, as well as detailed statistical results, have been uploaded as Supplementary Information to ensure full transparency and reproducibility.
